# Health systems readiness to provide geriatric friendly care services in Uganda: a cross-sectional study

**DOI:** 10.1186/s12877-019-1272-2

**Published:** 2019-09-18

**Authors:** Jude Thaddeus Ssensamba, Moses Mukuru, Mary Nakafeero, Ronald Ssenyonga, Suzanne N. Kiwanuka

**Affiliations:** 1 Makerere University College of Health Sciences, School of Public Health, Department of Health Policy and Planning, Kampala, Uganda; 2 Center for Innovations in Health Africa, Plot 1-3 School Road Namuwongo, P.O. Box 220, Kampala, Uganda; 3 Department of Epidemiology and Biostatistics, Makerere University School of Public Health, Kampala, Uganda

**Keywords:** Health facility readiness, Geriatric care, Uganda, health systems, Public health facilities, Africa

## Abstract

**Background:**

As ageing emerges as the next public health threat in Africa, there is a paucity of information on how prepared its health systems are to provide geriatric friendly care services. In this study, we explored the readiness of Uganda’s public health system to offer geriatric friendly care services in Southern Central Uganda.

**Methods:**

Four districts with the highest proportion of old persons in Southern Central Uganda were purposively selected, and a cross-section of 18 randomly selected health facilities (HFs) were visited and assessed for availability of critical items deemed important for provision of geriatric friendly services; as derived from World Health Organization’s Age-friendly primary health care centres toolkit. Data was collected using an adapted health facility geriatric assessment tool, entered into Epi-data software and analysed using STATA version 14. Kruskal-Wallis and Dunn’s post hoc tests were conducted to determine any associations between readiness, health facility level, and district.

**Results:**

The overall readiness index was 16.92 (SD ±4.19) (range 10.8–26.6). This differed across districts; Lwengo 17.91 (SD ±3.15), Rakai 17.63 (SD ±4.55), Bukomansimbi 16.51 (SD ±7.18), Kalungu 13.74 (SD ±2.56) and facility levels; Hospitals 26.62, Health centers four (HCIV) 20.05 and Health centers three (HCIII) 14.80. Low readiness was due to poor scores concerning; leadership (0%), financing (0%), human resources (1.7%) and health management information systems (HMIS) (11.8%) WHO building blocks. Higher-level HFs were statistically significantly friendlier than lower-level HFs (*p* = 0.015). The difference in readiness between HCIIIs and HCIVs was 2.39 (*p* = 0.025).

**Conclusion:**

There is a low readiness for public health facilities to provide geriatric friendly care services in Uganda. This is due to gaps in all of the health system building blocks. There is a need for health system reforms in Uganda to adequately cater for service provision for older adults if the 2020 global healthy ageing goal is to be met.

## Background

In 2017, the global population of older adults (aged 60 years and above) was 962 million. This is expected to rise to 2.1 billion by 2050, with 80% of them living in the developing world; Africa being home to 10.9% of them [1–3]. In response to the emminent challenge of an ageing population, the sixty-ninth World Health Assembly passed resolution 69.3, which advocated for a global strategy and plan of action on ageing and health [4]. The plan’s objectives were: to have a commitment from countries to act on healthy ageing, develop age-friendly environments, align health systems to the needs of the elderly, develop sustainable and equitable systems for geriatric care, and improve measurement, monitoring, and research for geriatric care [5].

There is evidence that Western countries have progressed well regarding the achievement of the objectives above [6]. However, there is a lack of information and research on the organisation and preparedness of public health systems in low-income countries, like Uganda, to offer geriatric friendly care services [7]. Moreover, such information is critical for policy decision making and financial allocative efficiency in the health sector, more so, if Africa is to meet the 2020 global healthy ageing goal [5].

### Uganda’s demographic and epidemiological transition

Uganda’s socio-economic transformation, internal security, and an improvement in health care services like maternal child health, immunisation, surgical care, and management of infectious diseases like HIV/AIDS, malaria, and respiratory tract infections [8], have seen an improvement in health indicators. According to the 2016 Uganda demographic health survey, childhood mortality rates declined from 87 deaths per 1000 live births in 1988 to 22 deaths per 1000 live births in 2016, maternal mortality rates per 100,000 live births declined from 524 mothers between 1994 and 2001 to 368 mothers between 2009 and 2016, over 87% of Ugandans know how to prevent themselves from acquiring HIV, and malaria prevalence is declining [9]. As a result, Uganda’s life expectancy has progressively increased past the 60-year-old mark, which the World Health Organisation defines as old age [5]. Relatedly, the number of Ugandans making it 60 years and above has progressively increased from 686,260 people, as per the 1991 Uganda Population and Housing Census, to 1,101,039 in 2002 [10] and 1,384,000 in 2014 [11]. This is expected to rise to above 5% of the national population by 2020 [12].

Relatedly, as the burden of infectious diseases declines, just like in other sub-Saharan African countries, there is a notable increase in the burden of non-communicable diseases in Uganda, more so among the older adults [13–16]. Although this could be attributed to the ageing process, changing lifestyles and the nutrition transition also have a role to play [14]. It is therefore pertinent that Uganda’s health care system is well prepared to handle the health needs of the elderly who are more prone to chronic non-communicable diseases.

### Geriatrics in the context of Uganda’s health system

Like other developing countries, Uganda’s public health system’s readiness to provide geriatric friendly services is not well documented [7]. Existing policy documents on old persons by the Ministry of Gender Labour and Social Development focus on only addressing their social needs [10]. Relatedly, The national health policy and Uganda’s vision 2040 policy documents do not address the need to institute health system changes focused on providing geriatric friendly health care services [17, 18], and this undermines the attainment of Sustainable Development Goal (SDG) 3 objectives that call for equitable health care access for all [19].

Geriatrics studies in Uganda have mainly focused on: exploring geriatric education needs, knowledge and attitudes of health workers towards geriatric care services (where 69% of them lacked geriatric training, and 80% had poor to fair knowledge about geriatric care [12]), the impact of HIV among older adults [20], older persons as caregivers to people infected and affected by HIV/AIDS [21], and loneliness among older adults in Uganda [22]. Other studies have focused on: health care access challenges faced by the elderly [23, 24], hyponatraemia among older adults [25], and perceptions of anaemia among the older population in Southwest Uganda [26].

### Assessing readiness for provision of geriatric friendly health care services

Readiness is the ability of a health facility to provide a given health service [27], and for geriatric care, the WHO’s 2008 Age-friendly Primary Health Care Centres Toolkit [28] provides an insight on which key systems and items that should be in place if a primary health care facility is to offer geriatric friendly care services. WHO’s “the measuring service availability and readiness health assessment (SARA) methodology for monitoring health systems strengthening” [29] and the USAID and health systems 20/20’s “The Health System Assessment Approach: A How-To Manual. Version 2.0” [30] have been developed for this purpose.

**Fig. 1:**
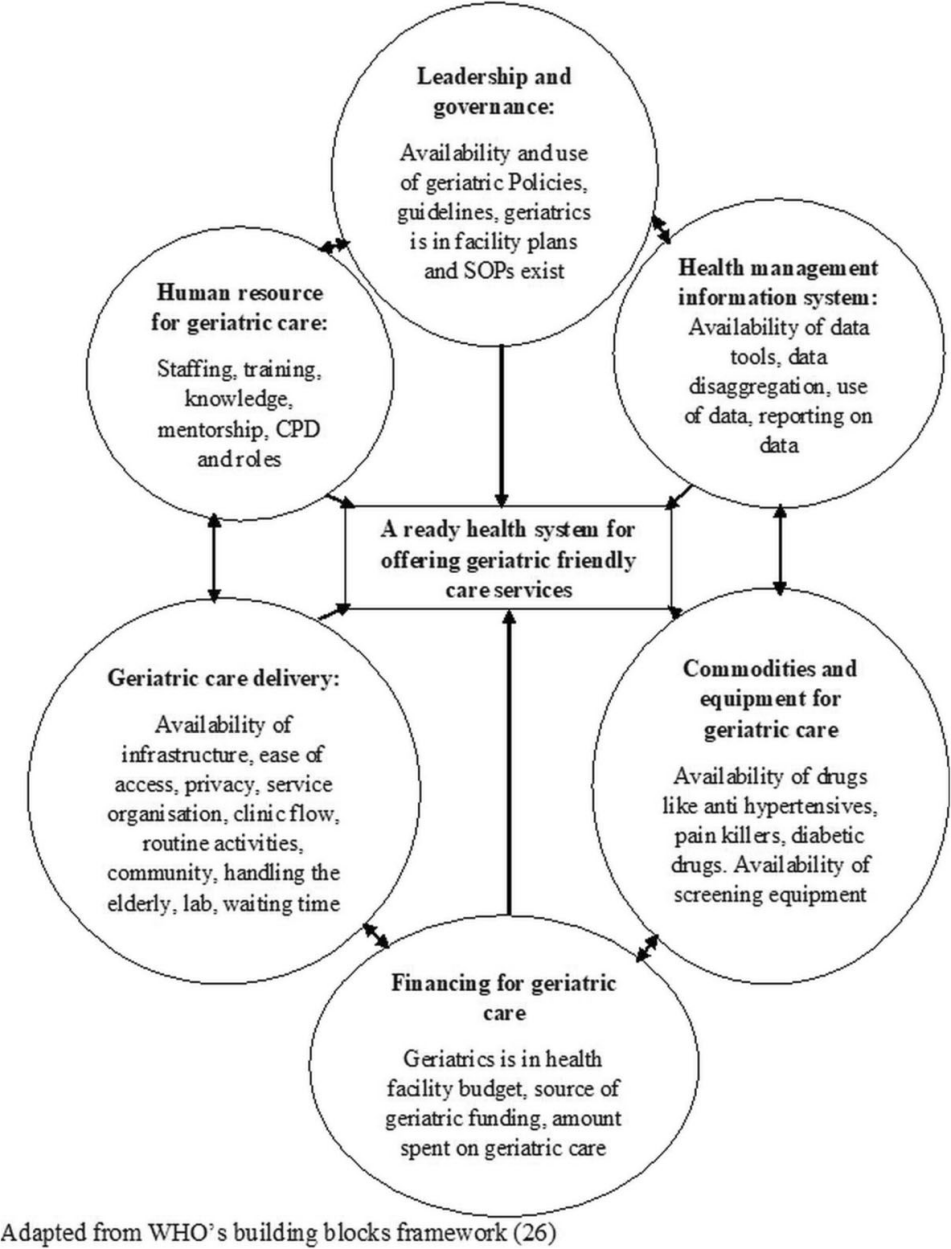
Study conceptual framework

The SARA methodology has effectively been used in resource-limited countries to assess: general health facility readiness [31], progress towards universal health coverage [32], maternal and child health services and non-communicable diseases in Bangladesh [33, 34], surgical services in Africa [35], and readiness of Ugandan health services for the management of outpatients with chronic diseases [36]. That said, based on a rigorous internet-based review of literature search, there are no studies that have been conducted to assess the readiness of public health facilities to provide geriatric friendly services in low- and middle-income countries. In the same line, the WHO building blocks approach enables an assessor to evaluate the six critical pillars for health service delivery: leadership and governance, health financing, health service delivery, human resources for health, medical products, logistics and technologies, and health management information systems (HMIS) [37] (Fig. 1).

Excellent leadership and governance are crucial for the institutionalisation of geriatric friendly care services because it is vital in developing policies and guiding documents, providing enabling environments and forging strategic partnerships with all key stakeholders [29, 38]. Furthermore, human resources for health are critical for geriatric service delivery [39]. In this case, the WHO recommends the essential availability of health staff with specialised geriatric training like geriatricians, geriatric nurses, and geriatric social workers [28]. Similarly, medical commodities and equipment complemented with an excellent service delivery setting are essential, not to mention financing which provides fluidity for the availability of all other WHO blocks, and HMIS which includes vital information for research and strategic decision making [5].

Assessing the readiness of public health systems to provide geriatric friendly care services is vital for countries to come up with policies that enable adequate planning for their ageing population. The objective of our study was to assess the readiness of public primary health care facilities to provide geriatric friendly services in Southern Central Uganda.

## Methods

### Study design and population

This cross-sectional study was conducted across public primary health care facilities located in four purposively selected districts of Bukomansimbi, Kalungu, Rakai and Lwengo which according to the 2014 National census data were home to the highest proportion of older adults; 5.9, 5.9, 5.0 and 4.8% respectively in Southern Central Uganda [8, 11, 40]. Presence of a high proportion of older adults in the selected districts was considered vital for understanding how health systems that assumedly interact more with these people were prepared to provide geriatric friendly services, while public health care facilities were selected because they provide health care to the majority of the rural population in Uganda [8], where most older adults dwell.

The study population was public primary health care facilities (HCIIIs, HCIVs, and district Hospitals) located within the four selected districts. HCIIIs are a first level referral HFs located at sub-county level and offer non-specialised preventive, curative, and promotive health care services. In Uganda, they serve an average population of 30,000 people and are headed by a Clinical Officer (Assistant Medical Officer). HCIVs are second-level referral health facilities located at the county level. On top of offering services offered at HCIIIs, they offer surgical, inpatient, and blood transfusion services. They are led by Medical Officers and are designed to serve 100,000 people. Hospitals are more specialised and are located at the district level. They offer more specialised health care services, are led by a medical director, and serve a population of over 200,000 people [17, 41]. Details on the organisation and complexity of Uganda’s public primary health care facilities have been described elsewhere [17, 42, 43].

### Sampling

We used the WHO’s workplace violence in the health sector sampling design frame [44] because it provides clear guidance on selecting primary health care facilities, and was easy to implement for this study. Here, HFs were stratified by district and level of HF. From each stratum, samples representing 30% or above of the population of HFs were randomly drawn. A total of 18 of 44 PHFs were selected. These included 12 of 36 HCIII, 5 of 6 HCIVs and 1 of 2 hospitals. The study was restricted to district level primary health care facilities [17]; excluding Health Centres two due to their low staffing norms and the government’s plan to abolish them, and regional and national referral hospitals that are tertiary care facilities.

### Data collection

Data was collected using a pretested geriatric health facility assessment tool whose variables were adapted from WHO’s 2008 Age-friendly Primary Health Care Centres Toolkit [28], USAID’s Health System Assessment Approach manual [30] and SARA [29]. The tool was designed following the WHO building blocks model. Data on a total of 103 variables (Additional file 1: Table S1) was collected, whereby heads of HFs or their delegates responded to closed questions. Observation, verification, and inspection were done to corroborate what the heads said. Collated data was entered into two passes of Epi-data version 4, compared for errors and exported to Stata version 14 for analysis. According to Uganda’s health system setup, items such as ambulances, X-ray machines, doctors, pharmacists, are not expected at HCIIIs, hence these were excluded from both the denominator and numerator during analysis for HCIIIs.

### Statistical analysis

#### Measuring readiness

We determined the readiness index (RI) for each HF based on SARA guidelines [29] by ascertaining percentage scores for variables within each WHO building block (See Additional file 1: Table S1). For each HF, the average of these scores was its RI. The mean of facility-level readiness indexes formed the overall RI (Additional file 2: Table S2).

RI = (a + b + c + d + e + f)/6 [29]. [Where a, b … f are mean scores for the six building blocks]. The overall RI was the mean of readiness indexes across all study sites.

To elicit associations between readiness and level and location of HFs; the Kruskal-Wallis test was run; while Dunn’s post hoc test determined which facility levels or districts differed in readiness.

#### Interpretation

For a HF to be deemed fully ready to provide geriatric friendly care services, it was expected to have an 80–100 RI score. Moderate readiness was a RI of 51–79, while low readiness was scoring 0–50.

## Results

Of the 18 HFs, 50% (*n* = 9) of them were from Rakai district, 22% (*n* = 4) from Lwengo district, three HFs (17%) from Kalungu district, and two HFs (11%) were selected from Bukomansimbi district. By facility level; 67% (*n* = 12) were HCIIIs, 28% (*n* = 5) HCIVs, and one hospital. The overall readiness index (RI) was 16.92 (range 10.8 to 26.6, SD 4.19). Of the four districts, Lwengo and Rakai districts had the highest readiness indexes of 17.91(SD 3.15) and 17.63 (SD 4.55) respectively, while Bukomansimbi and Kalungu districts had RIs of 16.51 (SD 7.18) and 13.74 (SD 2.56) respectively. The highest facility-level readiness index was at the hospital with a RI of 26.62, while HCIVs had a RI of 20.05 and HCIIIs a RI of 14.80 (Table 1). There was a significant difference in readiness indexes (*p* = 0.015) across HF levels, with Dunn’s post hoc test showing that the difference (2.39) was between HC IIIs and HC IVs (*p* = 0.025).

**Table 1:** Readiness index scores by district and health facility levels

Characteristics	Number of health units *n* (%)	Readiness indexes	Std. Dev. (±)
*Overall RI*	18 (100)	16.92	4.19
*District*			
Rakai	9 (50)	17.63	4.55
Bukomansimbi	2 (11)	16.51	7.18
Kalungu	3 (17)	13.74	2.56
Lwengo	4 (22)	17.91	3.15
*Health facility level*			
Health center III	12 (67)	14.80	2.34
Health center IV	5 (28)	20.05	3.22
Hospital	1 (5)	26.62	

### WHO building block scores as determinants of overall readiness

**Fig. 2:**
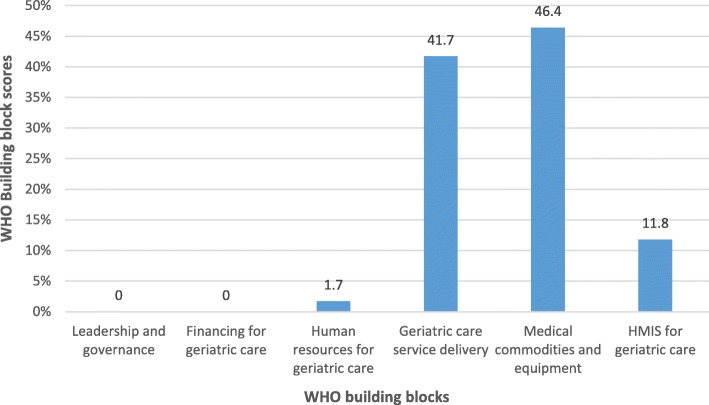
Mean WHO block-level scores at the 18 health facilities

Of the six WHO building blocks, the medical commodities, and equipment for geriatric care block had the highest score of 46.4, followed by the geriatric care services delivery block (41.7), HMIS for geriatric care (11.8), and human resource for geriatric care (1.7). The leadership and governance, and financing for geriatric care blocks had a score of zero (Fig. 2).

#### Leadership and governance for geriatric care

All HFs (*n* = 18) scored zero concerning this building block. They lacked geriatric care policies, national geriatric care management guidelines, had no geriatric focal personnel, and did not receive geriatric support visits. Relatedly, older adults were not represented on health unit committees and had no community networks to support them (Fig. 2).

#### Financing for geriatric care

Figure 2 shows that the score for this block was zero, attributable to all study HFs not having a work plan that incorporated geriatric care services into their routine care. Furthermore, there were no finances allocated to geriatric care activities, no HFs were receiving external funding for geriatric activities, and older adults did not receive any financial concessions for pay for services.

#### Human resource for geriatric care

The average block-level score was 1.7 (Fig. 2). Of the 18 HFs, only one HCIII (5.6%) had a geriatric health specialist, while a HCIV (5.6%) had a nurse that had some training in geriatric care. The rest of the health workers (HWs) had never had any geriatric training. Relatedly, no HFs were receiving mentorship in geriatrics, had plans to hire a geriatric specialist, or had personnel dedicated to supporting older adults (Table 2).

**Table 2:** Summary scores for select human resource for health tracer items at 18 HFs

Tracer item	Availability	HC III	HC IV	Hospital	Total
		Freq. (Col %)	Freq. (Col %)	Freq. (Col %)	
HF has geriatric care specialist^b^	Yes	1 (8.3)	0 (0)	0 (0)	1 (5.6)
	No	11 (91.7)	5 (100)	1 (100)	17 (94.4)
Doctor had geriatric care training	No	0 (0)	5 (100)	1 (100)	6 (100)
	NA	12 (0)^a^	0 (0)	0 (0)	
Clinical officer had geriatric training	Yes	1 (8.3)	0 (0)	0 (0)	1 (5.6)
	No	11 (91.7)	5 (100)	1 (100)	17 (94.4)
Nurses had geriatric training	Yes	0 (0)	1 (20)	0 (0)	1 (5.6)
	No	12 (100)	4 (80)	1 (100)	17 (94.4)
Midwives had geriatric training	No	12 (100)	5 (100)	1 (100)	18 (100)
The pharmacist had geriatric care training	No	1 (100)	5 (100)	1 (100)	7 (100)
	NA	11 (0)^a^	0 (0)	0 (0)	
Other health workers had geriatric training	No	12 (100)	5 (100)	1 (100)	18 (100)
The facility has a health worker to help older adults	No	12 (100)	5 (100)	1 (100)	18 (100)
The HF has support staff to help older adults	No	12 (100)	5 (100)	1 (100)	18 (100)
Health workers had geriatric training in the last two years	No	12 (100)	5 (100)	1 (100)	18 (100)
Staff receive CME in geriatric services	Yes	1 (8.3)	0 (0)	0 (0)	1 (5.6)
	No	11 (91.7)	5 (100)	1 (100)	17 (94.4)

^a^NA indicates not applicable for that level of HF and is not included in the analysis

^b^Indicates someone who had a geriatric-specific training that lasted 1 year or more

#### Geriatric care services delivery

The average score for this block was 41.7 (Fig. 2), attributable to all HFs (100%) having a waiting area, and 88.9% of them having a reception point accessible by older adults. Sixteen of 18 HFs were well lit and located within 5KM from the communities they serve. At Fifteen health units, their consultation rooms afforded privacy, while 12 HFs had their doors wide enough to allow for wheelchairs, and easy to open by older adults.

**Fig. 3:**
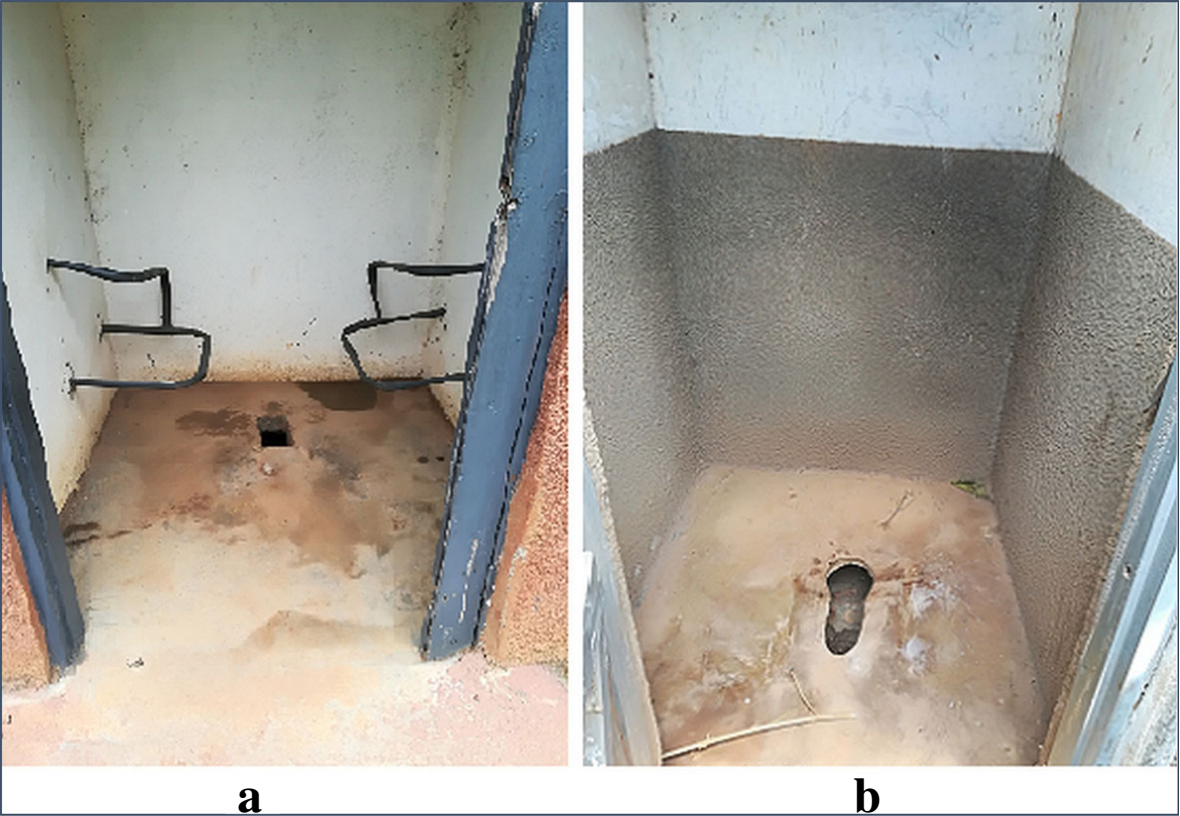
Type of toilets found at the majority of health facilities visited. A toilet with grab rails for old and disabled patients at one of the HFs (**a**), and a toilet without grab rails, standard at 16 of the visited HFs (**b**). *Photo by: Nayiga Maria© Center for innovations in Health Africa (CIHA, Uganda)*

On the other hand, all HFs lacked audio-visual information on geriatrics, only one HF was escorting old persons to points like the laboratory, and two HFs were providing health education (H/E) on ageing, with older adults allowed to ask questions. Only 2 HFs had toilets with grab rails (Fig. 3a), and only one HF had a special room for older adults. Only 4 HFs had information written in big reflective colours; easy for older adults to read. For other tracer items, see Table 3.

**Table 3:** Summary of scores for select geriatric care services delivery tracer items at 18 HFs

Tracer item	Availability	HC III *n* = 12	HC IV *n* = 5	Hospital *n* = 1	Total *n* = 18
		Freq. (Col %)	Freq. (Col %)	Freq. (Col %)	
Pathways at the HF are well paved with handrails and grabs	Yes	5 (41.70)	0 (0)	1 (100)	6 (35.29)
	No	7 (58.30)	5 (100)	0 (0)	12 (66.67)
HF markings readable by older adults	Yes	6 (50)	3 (60)	1 (100)	10 (55.6)
	No	6 (50)	2 (40)	0 (0)	8 (44.4)
Floors are rough to prevent falls	Yes	7 (58.3)	1 (20)	1 (100)	9 (50)
	No	5 (41.7)	4 (80)	0 (0)	9 (50)
Floor steps are simple for the old persons to climb	Yes	5 (62.5)	2 (66.67)	1 (100)	8 (66.67)
	No	3 (37.5)	1 (33.33)	0 (0)	4 (33.33)
	NA^a^	4 (0)	2 (0)	0 (0)	
Public transport readily available at the HF	Yes	8 (66.7)	5 (100)	1 (100)	14 (77.8)
	No	4 (33.3)	0 (0)	0 (0)	4 (22.2)
The HF gives priority to older adults	Yes	5 (41.7)	3 (60)	1 (100)	9 (50)
	No	7 (58.3)	2 (40)	0 (0)	9 (50)
Older adults are directed to key points	Yes	8 (66.7)	4 (80)	0 (0)	12 (66.7)
	No	4 (33.3)	1 (20)	1 (100)	6 (33.3)
HF has an equipped emergency resuscitation kit	Yes	0 (0)	5 (100)	1 (100)	6 (33.3)
	No	12 (100)	0 (0)	0 (0)	12 (66.7)
HF has an ambulance for referring patients	Yes	0 (0)	4 (80)	1 (100)	5 (8350)
	No	0 (0)	1 (20)	0 (0)	1 (17)
	NA^b^	12 (0)	0 (0)	0 (0)	

^a^NA indicates HF was constructed with no need for steps

^b^NA means not applicable for that level of HF and is not included in the analysis

#### Availability of diagnostics at visited health facilities

Table 4 shows that microscopy and urinalysis were conducted at 17 of the 18 HFs, and renal and liver function tests were conducted at six of the seven eligible HFs. On the other hand, across all districts, blood cholesterol, serum electrolytes, X-ray services, and ultrasound scan services were conducted at 1 of 6, 3 of 7, 1 of 6 and 4 of 6 eligible HFs respectively.

**Table 4:** Summary of scores for the availability of diagnostics at the 18 HFs

Sub-block item	Availability	HC III *n* = 12	HC IV *n* = 5	Hospital *n* = 1	Total *n* = 18
		Freq. (Col %)	Freq. (Col %)	Freq. (Col %)	
Microscopy	Yes	11 (91.7)	5 (100)	1 (100)	17 (94.4)
	No	1 (8.3)	0 (0)	0 (0)	1 (5.6)
Urinalysis	Yes	11 (91.7)	5 (100)	1 (100)	17 (94.4)
	No	1 (8.3)	0 (0)	0 (0)	1 (5.6)
Prostate surface antigen and tumour markers	No	0 (0)	5 (100)	1 (100)	6 (100)
	NA^a^	12 (0)	0 (0)	0 (0)	
Blood glucose or any other screening test for diabetes	Yes	5 (41.7)	5 (100)	1 (100)	11 (61.1)
	No	7 (58.3)	0 (0)	0 (0)	7 (38.9)
Visual acuity done at the HF	Yes	2 (16.7)	1 (20)	1 (100)	4 (22.2)
	No	10 (83.3)	4 (80)	0 (0)	14 (77.8)

^a^NA indicates not applicable for that level of HF and is not included in the analysis

#### Medical commodities and equipment for geriatric care

This WHO building block had the highest score of 46.4. All HFs (*n* = 18, 100%) had weighing scales and MUAC tapes, pain killers, anti-hypertensives, eye ointment, and antibiotics. Of the 18 HFs, blood pressure machines were available at 13 (72%), stethoscopes at 15 (83.3%), 11 HFs (61%) had glucometers, and 16 HFs (89%) had anti-malarial drugs. On the other hand, all HFs (*n* = 18) lacked hearing loss screening equipment, hearing aids, memory loss screening cards, and incontinence bags. Eyeglasses, walking crutches, white canes for the blind and other assistance devices for the blind were found at only one (5.6%) of the 18 HFs. Wheelchairs were only available at four (22%) HFs. For other tracer items, see Table 5.

**Table 5:** Summary of scores for select medical commodities and equipment tracer items at the 18 HFs

Tracer item	Availability	HC III	HC IV	Hospital	Total
		Freq. (Col %)	Freq. (Col %)	Freq. (Col %)	
Thermometer	Yes	4 (33.3)	5 (100)	0 (0)	9 (50)
	No	8 (66.7)	0 (0)	1 (100)	9 (50)
Visual acuity screening chart	Yes	2 (16.7)	1 (20)	1 (100)	4 (22.2)
	No	10 (83.3)	4 (80)	0 (0)	14 (77.8)
Wheel chairs	Yes	0 (0)	3 (60)	1 (100)	4 (22.2)
	No	12 (100)	2 (40)	0 (0)	14 (77.8)
Anti-diabetic drugs	Yes	0 (0)	5 (100)	1 (100)	6 (33.3)
	No	12 (100)	0 (0)	0 (0)	12 (66.7)
Nutrition supplements	Yes	3 (25)	1 (20)	1 (100)	5 (27.8)
	No	9 (75)	4 (80)	0 (0)	13 (72.2)
Antidepressants	Yes	10 (83.3)	4 (80)	1 (100)	15 (83.3)
	No	2 (16.7)	1 (20)	0 (0)	3 (16.7)
Anticholinergic drugs for incontinence	Yes	1 (8.3)	2 (40)	0 (0)	3 (16.7)
	No	11 (91.7)	3 (60)	1 (100)	15 (83.3)
Benzodiazepines for insomnia	Yes	10 (83.3)	5 (100)	1 (100)	16 (88.9)
	No	2 (16.7)	0 (0)	0 (0)	2 (11.1)
Oxygen cylinders	Yes	0 (0)	4 (80)	1 (100)	5 (27.8)
	No	12 (100)	1 (20)	0 (0)	13 (72.2)

#### Health management information systems for geriatric care

The overall score for this block was 11.8 (Fig. 2). All HFs (*n* = 18, 100%) had out-patient department (OPD) registers that segregated data by age at all HFs (*n* = 18, 100%), and 17 of them (94.4%) were reporting on geriatric data through DHIS2. On the other hand, all HFs lacked vital tools: the geriatric medical assessment tool, geriatric comprehensive screening tool, geriatric mental state examination tool, memory loss evaluation form, geriatric depression scale, urinary incontinence evaluation form, fall evaluation form, and geriatric daily activity form. No HF was using geriatric data to improve service delivery (Table 6).

**Table 6:** Showing a summary of scores for HMIS tracer items at the 18 HFs

Tracer item	Availability	HC III *n* = 12	HC IV *n* = 5	Hospital *n* = 1	Total
		Freq. (Col %)	Freq. (Col %)	Freq. (Col %)	
OPD registers with age disaggregation	Yes	12 (100)	5 (100)	1 (100)	18 (100)
Inpatient registers with age disaggregation	No	12 (100)	5 (100)	1 (100)	18 (100)
HIV registers with age disaggregation	No	12 (100)	5 (100)	1 (100)	18 (100)
Laboratory registers with age disaggregation	No	12 (100)	5 (100)	1 (100)	18 (100)
Other registers with age disaggregation	No	12 (100)	5 (100)	1 (100)	18 (100)
Geriatric daily activity form	No	12 (100)	5 (100)	1 (100)	18 (100)
HF collects geriatric care data	No	12 (100)	5 (100)	1 (100)	18 (100)
Data at facility is segregated by age	Yes	0 (0)	0 (0)	1 (100)	1 (5.6)
	No	12 (100)	5 (100)	0 (0)	17 (94.4)
HF reports geriatric data through DHIS2	Yes	11 (91.7)	5 (100)	1 (100)	17 (94.4)
	No	1 (8.3)	0 (0)	0 (0)	1 (5.6)
HF running any geriatric-focused project for which data is utilised	No	12 (100)	5 (100)	1 (100)	18 (100)

### WHO building block scores by HF level

**Fig. 4:**
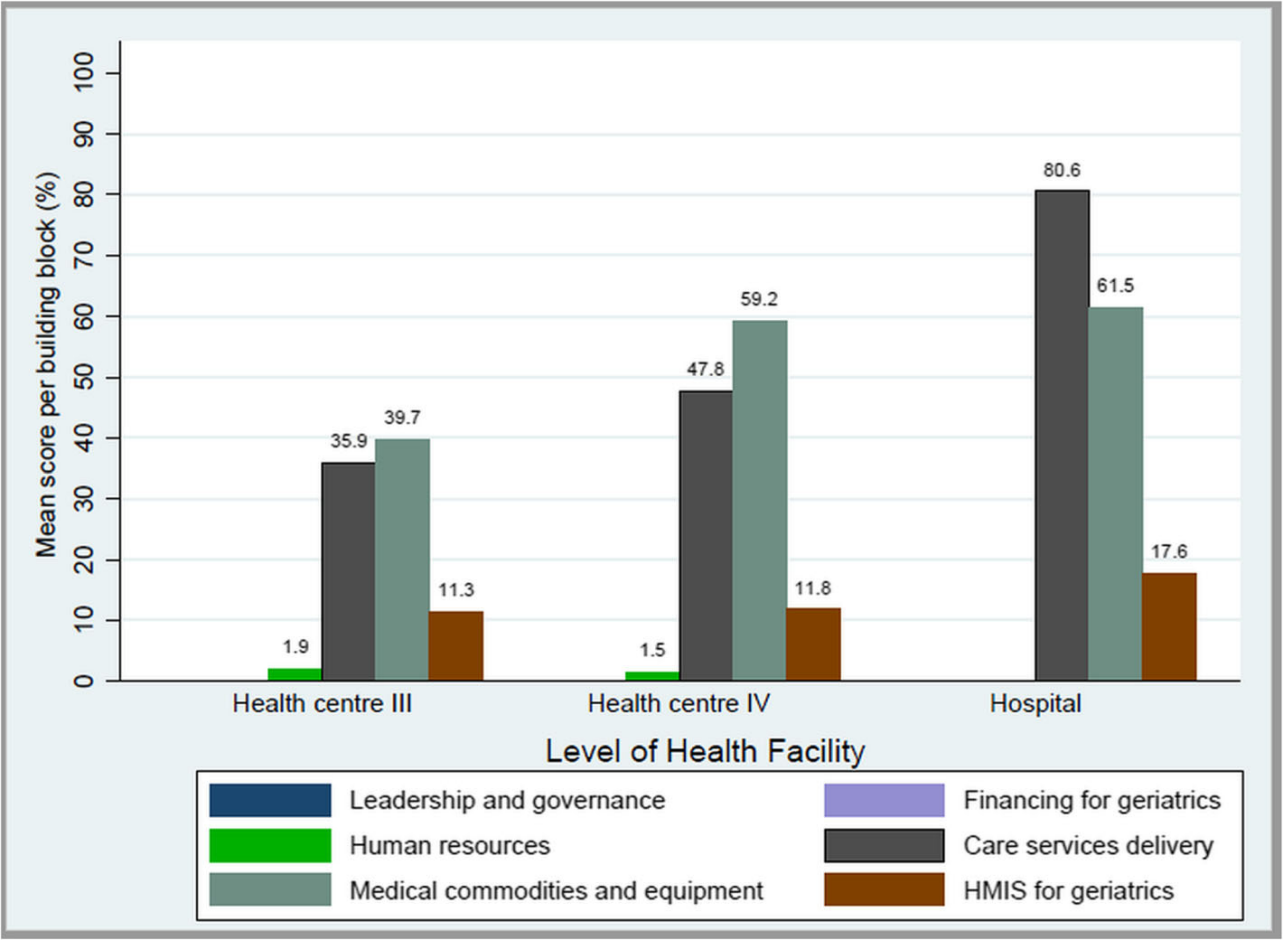
WHO building block scores by level of health facility

The hospital had the highest block-level ratings: 80.6 for care service delivery, and 61.5 for medical commodities and equipment, and 17.6 for HMIS. HCIVs scored 47.8, 59.2, 11.8 and 1.5 for care service delivery, medical commodities and equipment, HMIS, and human resource for geriatric care respectively. HCIIIs had the least scores of: 35.9, 39.7, 11.3, and 1.9 for care service delivery, medical commodities and equipment, HMIS, and human resource for geriatric care respectively (Fig. 4).

### WHO building block scores by district

**Fig. 5:**
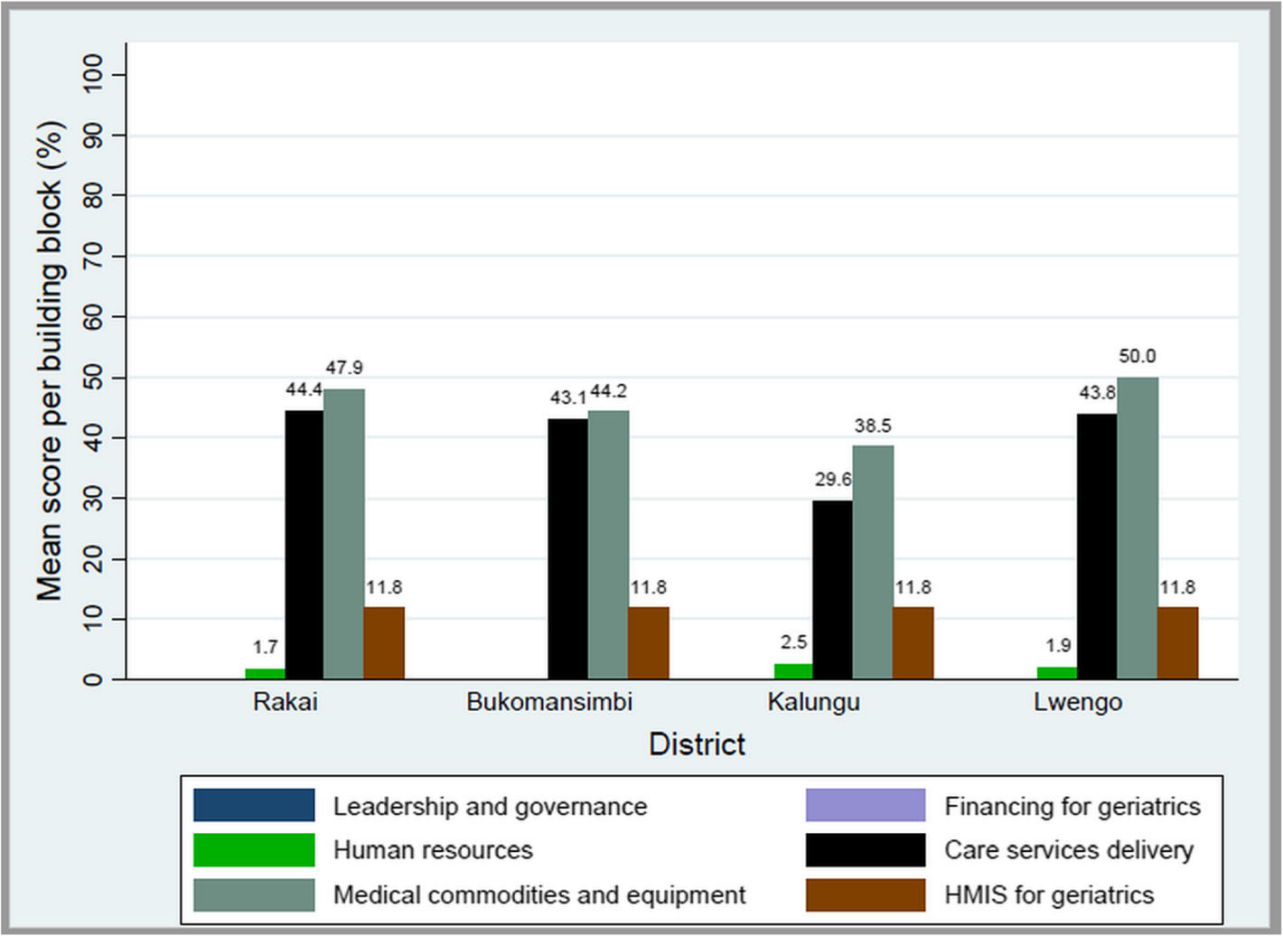
WHO building block scores by district

Lwengo district had the highest WHO block-level scores: 50, 43.8, 11.8 and 1.92 for medical commodities and equipment, care service delivery, HMIS, and human resource for geriatric care respectively. Rakai district had scores of 47.9, 44.4, 11.8 and 1.71 for medical commodities and equipment, care service delivery, HMIS, and human resource for geriatric care respectively, Bukomansimbi scored: 44.2, 43.1, and 11.8 for medical commodities and equipment, care service delivery and HMIS respectively, while Kalungu had the least scores of 38.5 for commodities and equipment, 29.6 for care service delivery, 11.8 for HMIS, and 2.56 for human resources for geriatrics (Fig. 5).

## Discussion

This study which to our knowledge is the first published endeavour investigated the readiness of Uganda’s public health facilities to provide geriatric friendly care services. The overall readiness index was low (16.92) across all study districts and HFs, though hospitals (26.62) and HCIVs (20.05, SD = 3.22) were more prepared compared to HCIIIs (14.80, SD = 2.34) (Table 2). The overall WHO block-level readiness scores were relatively high in regard to geriatric care service delivery (41.4) and medical commodities and equipment for geriatric care (46.4) compared to HMIS (11.8), human resource for geriatric care (1.7), leadership and governance (0), and financing for geriatric care (0).

Our findings concur, with existing literature, that African health care systems are still far from providing geriatric friendly care services to their ageing population [7]. In Uganda’s context, although no studies have looked at the full scale of the state of geriatric care services at public health facilities, factors for low readiness identified in this study have been documented in other studies. These highlighted that Uganda lacks specialised geriatric centers [45], trained geriatric specialists, geriatric training institutions [12] and that older adults face health care access challenges [23]. For this, a system-wide response is vital if Uganda is to achieve the 2020 global health ageing goal [5].

The significant difference in readiness indexes (*p* = 0.015) across HF levels, where hospitals were more ready (26.62) compared to HCIVs (20.05, SD 3.22) and HCIII (14.80, SD 2.34), has been documented in other health systems assessment studies conducted in Uganda. For example, Katende et al., (2015), found hospitals and HCIVs to more equipped with regard to the availability of essential supplies, training, drugs, and diagnostic equipment as compared to hospitals and HCIVs [36] for management of outpatients with chronic diseases. The above could be due to Uganda’s health system organisation which equips higher level HFs with more specialised equipment, commodities, finances, and HWs compared to lower-level community-based HFs [8, 41, 42]. It, however, raises access challenges given older adults have to travel long distances as higher-level HFs tend to be located further from their residences [23]. It is vital that lower-level HFs are equipped too, given that they are closer to communities, and serve where most older adults dwell.

### WHO building blocks and readiness for geriatric friendly services

Absence of critical documents like geriatric policies, geriatric management guidelines and lack of leadership and partnerships for geriatric care across all 18 HFs resonates with existing literature that Africa is ill-equipped to address the health needs of its rising ageing population [7, 46]. Moreover, leadership and governance are key for providing strategic direction and an enabling environment for service delivery [38]. The Global Strategy and Action Plan on Ageing and Health call for leaders and policymakers to institutionalise geriatric care into their health systems [5]; more so if SDG number 3 which calls for equitable good health and well-being of all people [19] is to be achieved. It is therefore pertinent that a framework for the institutionalisation of geriatric care in Uganda’s health system is introduced.

Inadequate funding for geriatric care has already been noted in sub-Saharan Africa [7], and our findings further validate Uganda’s case. However, this could be attributed to a system-wide gap where the health sector in Uganda is allocated lesser finances compared to other areas like roads and defence [8]. Based on current literature, Uganda is yet to meet the Abuja declaration target of allocating 15% of its financial resources to health care [47–49] which leaves many gaps in the provision of care to its masses. For Uganda to meet the health needs of older adults, increased budgetary allocation to the health sector is key.

Our findings showed that the majority of HWs lacked adequate training and skills in the provision of geriatric care. This is consistent with a study which found that 69% of rural health workers lacked geriatric training, and 80% of them had poor to fair knowledge of geriatrics [12]. Furthermore, our findings validate the assertion that African countries have made little progress in training its health workers in geriatric care [46]. For Uganda, this could be attributed to the absence of health training institutions that conduct training and have specialisations in geriatrics [12]. Relatedly, the fact that no HF has ever had a CME or mentorship on geriatrics, that have been found to improve knowledge, skills, and practice among HWs [12, 50], highlights an urgent need for action if Uganda is to meet the 2020 global healthy ageing goal.

The reasonably good geriatric service delivery block score (41.5) can be attributed to the Government of Uganda’s current efforts to improve its health system through making health facilities more accessible and equipped with laboratory sundries, and screening equipment [8]. On the other hand, identified gaps like inadequacy of audio-visual information on geriatrics, special rooms for older adults, friendly toilets and information written in sizes and colours easily read by older adults, show that sub-Saharan Africa continues to lag with regard to organisation for provision of geriatric care [7], though it provides an opportunity for action if Uganda is to align with the global healthy ageing strategy.

The highest WHO block-level score was with the medical commodities and equipment for geriatric care block (46.4). This is attributable to policies that have led to system improvements at the National Medical Store; Uganda’s autonomous body responsible for procurement and supply of medical equipment and medicines across all public health facilities in Uganda [51]. However, essential equipment and commodities for conditions prevalent among the older adults like eyeglasses, hearing aids, incontinence bags, memory loss screening cards, crutches, and wheelchairs, were noticeably absent at the majority of HFs. It is critical that such items are provided at community level HFs to ensure that the older adults’ quality of life is improved.

The low HMIS block score (11.8) correlates with existing data that HMIS remains a challenge in Africa [52] and Uganda [53–55]. The absence of tools like the geriatric medical assessment tool, geriatric comprehensive screening tool, geriatric mental state examination tool, memory loss evaluation form, and Geriatric Depression Scale (GDS at all the 18 HFs raises questions on proper management of geriatric health conditions and use of data for decision making. It is thus crucial that Uganda’s HMIS is aligned to strategic objective five of the global strategy and plan of action on ageing and health; that calls on governments to institute monitoring and evaluation and HMIS systems to track geriatric indicators that are key for planning [4, 5].

### Strengths

To our knowledge, this study provides the first published insight on the readiness of public health facilities to offer geriatric friendly care services in Uganda and Africa, thus providing useful information for policy action. Furthermore, our study utilised data from different districts and levels of HFs; making findings more generalizable. During data collection, we used standardised piloted tools and triangulation through observation and verification. Lastly, the tools used for data collection were adapted from published, internationally proven documents like SARA [29], the Health Systems Approach Manual [30] and the Age-friendly primary health care centers toolkit [28].

### Limitations

Our study was restricted to public primary HFs, leaving out regional, national, private, and private not for profit HFs. This leaves an information gap on geriatric readiness in other health service sectors. Furthermore, the study was limited to districts in Central Uganda which could affect country-level generalisation. However, efforts were made to integrate a diverse group of HFs across various districts and locations to enrich the findings. Lastly, the two hospitals in this study were located within one district. For our study sample, we chose one hospital, which made eliciting associations at that level hard. We recommend country-based surveys that include non-public health facilities, further validation of the geriatric primary health care facility assessment tool, and anthropological studies to augment our findings.

## Conclusions

These findings indicate that Uganda’s public primary health care system is not adequately ready to provide geriatric friendly care services. This is due to gaps in leadership and governance, financing, human resource, HMIS and equipment and commodities for geriatric care. For Uganda to align with the 2020 global healthy ageing goal, changes in policy, financing, human resource for geriatric care, creating a favourable environment for older adults and HMIS for geriatrics need improvement, more so at lower level community HFs where the majority of older adults seek care.

## Supplementary information

**Additional file 1: Table S1.** The geriatric readiness score framework.

**Additional file 2: Table S2.** A guide for calculating HF block level scores.

## Data Availability

The datasets generated and/or analysed during the current study are not publicly available due to ethical requirements by the ethics committee but are available from the corresponding author on reasonable request.

## Abbreviations

AIDS: Acquired immunodeficiency syndrome
CME: Continuing Medical Education
DHIS2: District Health Information Systems 2
HCIII: Health Centre three
HCIV: Health Centre four
HF: Health Facility
HFs: Health Facilities
HIV: Human Immunodeficiency Virus
HMIS: Health Management Information System
HWs: Health workers
MoH: Ministry of Health
MUAC: Mid-upper arm circumference
OPD: Outpatient department
PHFs: Public health facilities
RI: Readiness index
SARA: Service Availability and Readiness health Assessment
SDG: Sustainable Development Goals
UBOS: Uganda Bureau of Statistics
UN: United Nations
USAID: United States Development Agency
WHO: World Health Organisation
