# Supplementary material for: Health systems readiness to provide geriatric friendly care services in Uganda: a cross-sectional study
Source: BMC Geriatr. 2019 Sep 18;19:256. doi: 10.1186/s12877-019-1272-2 (PMC6749715; doi:10.1186/s12877-019-1272-2)
Supplement: Supplementary file 1 — Additional file 1: Table S1. The geriatric readiness score framework. [file 12877_2019_1272_MOESM1_ESM.docx]

**Table S1**: **The geriatric readiness score framework**

| **Building block** | **Sub-block** | **Tracer items** | **Sub-block score** |
| --- | --- | --- | --- |
| Leadership and governance  (a) | Guiding documents for geriatric care (a1) | The geriatric policy (a1.1) | (n/2)*100 |
|  |  | Geriatric mgmt. guidelines (a1.2) |  |
|  | Leadership for geriatric care (a2) | Clinic has a geriatric FP (a2.1) | (n/2)*100 |
|  |  | The clinic has a supervisor for geriatric care services (a2.2) |  |
|  | Stakeholders and partners for geriatric care (a3) | Old persons represented on health unit committee (a3.1) | (n/3)*100 |
|  |  | A community network for older adults (a3.2) |  |
|  |  | An external partner that supports geriatric care at the HF (a3.3) |  |
| Financing for geriatric care services  (b) | Are geriatric care activities incorporated into the HF work plan? (b1) | HF work plan incorporates geriatric care activities (b1.1) | (n/4)*100 |
|  |  | The HF budget includes allocations to geriatric activities (b1.2) |  |
|  |  | The HF receives external financial support for geriatrics (b1.3) |  |
|  |  | Older adults receive financial health service incentives (b1.4) |  |
| Human resource for geriatric care services (c) | Training for geriatric care delivery (c1) | The HF has trained geriatric care specialist (c1.1) | (n/8)*100 |
|  |  | The doctor had some geriatric care training (c1.2) |  |
|  |  | The clinical officer had some geriatric care training (c1.3) |  |
|  |  | Nurses had some geriatric care training (c1.4) |  |
|  |  | Midwives had some geriatric care training (c1.5) |  |
|  |  | Pharmacists had some geriatric care training (c1.6) |  |
|  |  | Other health workers had some geriatric care training (c1.7) |  |
|  |  | The HF plans to hire a geriatric practitioner (c1.8) |  |
|  | Support for older adults(c2) | The HF has a health worker to help older adults (c2.1) | (n/2)*100 |
|  |  | The HF has support staff to help out older adults (c2.2) |  |
|  | Continuous professional development and support in geriatric care (c3) | Health workers received geriatric care training in the last two years (c3.1) | (n/3)*100 |
|  |  | HF staff receive CPD in geriatric care (c3.2) |  |
|  |  | HF receives mentorship in geriatric care (c3.3) |  |
| Geriatric care service delivery (d) | A geriatric friendly physical HF (d1) | Pathways at the HF well paved with handrails and grabs (d1.1) | (n/10)*100 |
|  |  | HF markings easily read by old persons (d1.2) |  |
|  |  | Reception easily accessible to older adults (d1.3) |  |
|  |  | Floors rough to prevent falls (d1.4) |  |
|  |  | Doors wide enough to allow for wheelchairs and easy to open by older adults (d1.5) |  |
|  |  | Toilets designed to accommodate old person’s needs (d1.6) |  |
|  |  | Floor steps at HF simple for the old to recognize and climb (d1.7) |  |
|  |  | HF well-lit for ease of movement by older adults (d1.8) |  |
|  |  | The HF has a waiting area for the aged (d1.9) |  |
|  |  | Reception area fitted with printed and audio-visual information on geriatric care (d1.10) |  |
|  | Public access to the HF (d2) | The HF is not more than 5 kilometres from the community it serves (d2.1) | (n/2)*100 |
|  |  | Public transport readily available at or nearby the HF (d2.2) |  |
|  | Privacy for older adults (d3) | Consultation rooms lockable (d3.1) | (n/3)*100 |
|  |  | Screens available (d3.2) |  |
|  |  | Inpatient older adults have special rooms at the HF (d3.3) |  |
|  | Assistance services for the aged (d4) | The clinic has a reception where older adults can easily inquire (d4.1) | (n/6)*100 |
|  |  | The HF gives priority to the old (d4.2) |  |
|  |  | The aged are appropriately directed to key points like the laboratory (d4.3) |  |
|  |  | Older adults are escorted by someone to key health points (d4.4) |  |
|  |  | The HF runs a special geriatric clinic or unit (d4.5) |  |
|  |  | All key information is written in big and reflective colours for easy reading by old persons (d4.6) |  |
|  | Education on geriatric care and information materials (d5) | Health workers talk about geriatric care during health education (d5.1) | (n/3)*100 |
|  |  | Older adults are given the opportunity to ask questions during health education sessions (d5.2) |  |
|  |  | The HF has information leaflets on ageing and health (d5.3) |  |
|  | Handling geriatric emergencies (d6) | An equipped emergency resuscitation kit is in place (d6.1) | (n/2)*100 |
|  |  | The HF has an ambulance to refer (d6.2) |  |
|  | Investigative services for older adults (d7) | Microscopy (d7.1) | (n/10)*100 |
|  |  | Urinalysis (d7.2) |  |
|  |  | Renal and liver function tests (d7.3) |  |
|  |  | Prostate surface antigens and tumour markers (d7.4) |  |
|  |  | Blood cholesterol (d7.5) |  |
|  |  | Blood glucose (or any other screening tests for diabetes) (d7.6) |  |
|  |  | Serum electrolytes (d7.7) |  |
|  |  | X-ray machines (d7.8) |  |
|  |  | Ultrasound scan (d7.9) |  |
|  |  | Is visual acuity done at the HF (d7.10) |  |
| Medical commodities and equipment for geriatric care (e) | Equipment for provision of geriatric services (e1) | Weighing scale or adult MUAC tapes for nutrition assessment (e1.1) | (n/8)*100 |
|  |  | Blood pressure machine (e1.2) |  |
|  |  | Thermometer (e1.3) |  |
|  |  | Glucometer (e1.4) |  |
|  |  | Stethoscope (e1.5) |  |
|  |  | Visual acuity screening chart (e1.6) |  |
|  |  | Hearing screening equipment (e1.7) |  |
|  |  | Memory loss screening cards (e1.8) |  |
|  | Commodities for geriatric care (e2) | Incontinence bags (e2.1) | (n/7)*100 |
|  |  | Eyeglasses (e2.2) |  |
|  |  | Hearing aids (e2.3) |  |
|  |  | Walking clutches (e2.4) |  |
|  |  | Walking aids for the blind (e2.5) |  |
|  |  | Wheelchairs (e2.6 |  |
|  |  | Any other assistance devices (e2.7) |  |
|  | Drugs for management of common geriatric health conditions (e3) | Pain killers like paracetamol (e3.1) | (n/11)*100 |
|  |  | Anti-hypertensive drugs (e3.2) |  |
|  |  | Anti-diabetic drugs (e3.3) |  |
|  |  | Eye drops and ointments (e3.4) |  |
|  |  | Antibiotics (e3.5) |  |
|  |  | Nutrition supplements (e3.6) |  |
|  |  | Antidepressants (e3.7) |  |
|  |  | Anticholinergic drugs for incontinence (e3.8) |  |
|  |  | Anti-malarial drugs (e3.9) |  |
|  |  | Benzodiazepines for insomnia management (e.g. Temazepam) (e3.10) |  |
|  |  | Oxygen (e3.11) |  |
| Health management information systems for geriatric care (f) | National HMIS tools sensitive to geriatric care (f1) | OPD register (f1.1) | (n/5)*100 |
|  |  | Inpatient register (f1.2) |  |
|  |  | HIV registers (f1.3) |  |
|  |  | Laboratory registers (f1.4) |  |
|  |  | Other registers (f1.5) |  |
|  | Availability of geriatric assessment tools (f2) | Geriatric medical assessment tool (f2.1) | (n/8)*100 |
|  |  | Geriatric comp. screening tool (f2.2) |  |
|  |  | Geriatric mental state examination tool (f2.3) |  |
|  |  | Memory loss evaluation form (f2.4) |  |
|  |  | Geriatric Depression Scale (GDS) (f2.5) |  |
|  |  | Urinary incontinence evaluation form (f2.6) |  |
|  |  | Falls evaluation form (f2.7) |  |
|  |  | Geriatric daily activity form (f2.8) |  |
|  | Data for service improvement (f3) | HF collects geriatric care data (f3.2) | (n/2)*100 |
|  |  | Data is segregated by age (f3.2) |  |
|  | Data reporting (f4) | HF reports through DHIS2 (f4.1) | (n/1)*100 |
|  | Research on ageing (f5) | HF running any geriatric-focused project for which data is utilized (f5.1) | (n/1)*100 |
| Note: “n” is the total number of tracer items noted to be available at the HF per sub-block | | | |

Calculations adapted from the SARA tool ([24](#_ENREF_24)).
