# Supplementary material for: Health systems readiness to provide geriatric friendly care services in Uganda: a cross-sectional study
Source: BMC Geriatr. 2019 Sep 18;19:256. doi: 10.1186/s12877-019-1272-2 (PMC6749715; doi:10.1186/s12877-019-1272-2)
Supplement: Supplementary file 2 — Additional file 2: Table S2. A guide for calculating HF block level scores. [file 12877_2019_1272_MOESM2_ESM.docx]

**Table S2: A guide for calculating HF block level scores**

| **Building block** | **Sub-block** | **Sub-block score** | **Building block score** |
| --- | --- | --- | --- |
| Leadership and governance  (a) | Guiding documents for geriatric care (a1) | a1 = [(∑a1.1… a1.2)/2]*100 | a=(a1+a2+a3)/3 |
|  | Leadership for geriatric care (a2) | a2 = [(∑a2.1… a2.2)/2]*100 |  |
|  | Stakeholders and partners for geriatric care (a3) | a3 = [(∑a3.1… a3.3)/3]*100 |  |
| Financing for geriatric care services (b) | Are geriatric care activities incorporated into the HF work plan? (b1) | b1 = [(∑b1.1… b1.4)/4]*100 | b=b1 |
| Human resource for geriatric care services (c) | Training for geriatric care delivery (c1) | c1 = [(∑c1.1… c1.8)/8]*100 | c=(c1+c2+c3)/3 |
|  | Support for older adults (c2) | c2= [(∑c2.1… c2.2)/2]*100 |  |
|  | Continuous profession development and support in geriatric care (c3) | C3 = [(∑c3.1… c3.3)/3]*100 |  |
| Geriatric care service delivery (d) | A geriatric friendly physical HF (d1) | d1 = [(∑d1.1… d1.10)/10]*100 | d=(d1+d2+d3+d4+d5+d6+d7)/7 |
|  | Public access to the HF (d2) | d2 = [(∑d2.1… d2.2)/2]*100 |  |
|  | Privacy for older adults (d3) | d3 = [(∑d3.1… d3.3)/3]*100 |  |
|  | Assistance services for older adults (d4) | d4 = [(∑d4.1… d4.6)/6]*100 |  |
|  | Education on geriatric care and information materials (d5) | d5 = [(∑d5.1… d5.3)/3]*100 |  |
|  | Handling geriatric emergencies (d6) | d6 = [(∑d6.1… d6.2)/2]*100 |  |
|  | Investigative services for older adults (d7) | d7 = [(∑d7.1… d7.10)/10]*100 |  |
| Medical commodities and equipment for geriatric care (e) | Equipment for provision of geriatric services (e1) | e1 = [(∑e1.1… e1.8)/8]*100 | e=(e1+e2+e3)/3 |
|  | Commodities for geriatric care (e2) | e2 = [(∑e2.1… e2.7)/7]*100 |  |
|  | Drugs for mgmt. of common geriatric health conditions (e3) | e3 = [(∑e3.1… e3.11)/11]*100 |  |
| Health management information systems for geriatric care (f) | National HMIS tools sensitive to geriatric care (f1) | f1 = [(∑f1.1… f1.5)/5]*100 | f=(f1+f2+f3+f4+f5)/5 |
|  | Availability of geriatric assessment tools (f2) | f2 = [(∑f2.1… f2.8)/8]*100 |  |
|  | Data for geriatric service improvement (f3) | f3 = [(∑f3.1… f3.2)/2]*100 |  |
|  | Reporting on geriatric data (f4) | f4 = [(∑f4.1… f4.1)/1]*100 |  |
|  | Research on ageing (f5) | f5 = [(∑f5.1… f5.1)/1]*100 |  |
